# A three-year-old boy with hypodipsic hypernatremia syndrome

**DOI:** 10.11604/pamj.2018.30.250.12263

**Published:** 2018-08-06

**Authors:** Bousayna Iraqi, Rachid Abilkassem, Nezha Dini, Aomar Agadr

**Affiliations:** 1 Department of Paediatrics, Mohammed V Military Hospital, Mohammed V University, Rabat, Morocco

**Keywords:** Brain calcifications, child, hypodipsic hypernatremia syndrome

## Abstract

We describe a three-year-old boy who had a growth and psychomotor retardation associated with inappropriate lack of thirst and vasopressin secretion in the presence of chronic plasma hyperosmolarity. Computed brain tomography revealed bilateral supratentorial sub-ependymal and cortical calcifications. Dissociation in the plasma vasopressin response to osmotic change was demonstrated in this patient. Treatment with a vasopressin analogue, desamino-D-arginine vasopressin (DDAVP) and forced intake of water restored plasma osmolality and serum sodium levels to normal.

## Introduction

A dipsic hypernatremia is an uncommon disorder caused by a defect in the osmoregulation of thirst, leading to chronic hyperosmolarity of body fluids [1]. The syndrome is characterized by chronic or recurrent episodes of severe hypernatremia associated with dehydration and lack of thirst. Often, the defect in secretion of vasopressin is clinically inapparent and can be demonstrated only by assay of the hormonal response to osmotic stimulation or suppression [2]. We describe the case of a Moroccan boy with inappropriate lack of thirst and vasopressin secretion in the presence of chronic plasma hyperosmolarity.

## Patient and observation

A three-year old boy came to our department with the chief complaint of growth and psychomotor retardation. He was born at term via spontaneous, uncomplicated vaginal delivery to a healthy 25-year-old primigravida. His birthweight was 3.5 kg (90 percentile), length was 49cm (50 percentile) and head circumference was 34cm (75 percentile). He is an only child of non-consanguineous parents. On admission, the mother indicated that since the age of one year, her son presented a psychomotor retardation: he could not sit; he could not stand tall and has been drinking very little. No histories of similar complaints in the family were noted. On arrival, physical examination reveals a weight of 12 kg (-2SD), a height of 88 cm (-2SD) and he presented a microcephaly with a head circumference of 46 cm (-2SD). There were no facial dismorphology, no hypopigmented macules and no dehydration signs. Examination of his mental status revealed that the child was alert, active and able to follow most verbal commands but he could not speak. He followed visual stimuli, and the pupils were equal in size and reactive to light. He moved all extremities purposefully and with full strength. There was ankle clonus with extensor plantar responses bilaterally, but he could not stand up without help and was unable to walk.

**Figure 1:**
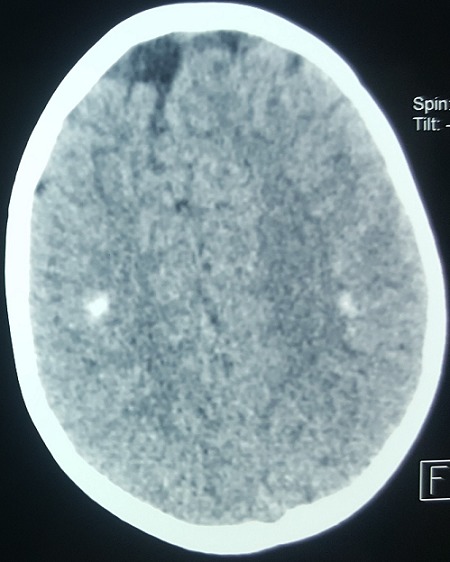
Cranial CT scan demonstrating bilateral supra-tentorial and sub-ependymal and cortical calcifications

The sensory examination was normal. Laboratory investigations on admission revealed that the serum electrolytes are: sodium, 167 mmol/l; potassium, 3.3 mmol/l, chloride, 138 mmol/l, and bicarbonate, 26 mmol/l; BUN (blood urea nitrogen) is 0.75 mg/dl, creatinine, 8mg/l; glucose, 0.80g/l and plasma osmolality 310 mmol/kg (normal range 280 to 295mmol/kg). The CSF has 2 WBCs and normal concentrations of glucose and protein. Urinalysis showed an osmolality of 523 mosml/l (normal range 300-900). Neither glucosuria nor proteinuria was noted. Results of a complete blood count, platelet count, measurement of the erythrocyte sedimentation rate were normal. Total plasma calcium, phosphorus, ionized calcium, alkaline phosphatase, parathyroid hormone and 25-hydroxyvitamin D2 were all within normal limits. Adipsic hypernatremia was suspected, and the patient underwent an extensive evaluation of the anterior and posterior portions of the hypophysis, with special interest in vasopressin secretion. Levels of serum prolactin and thyroid hormones as well as the thyrotropin response to thyrotropin releasing hormone were all within the normal range, as were growth hormone, cortisol and gonadotropin response to gonadotropin-releasing hormone. Computed tomography of the brain revealed bilateral supratentorial sub-ependymal and cortical calcifications (Figure 1). Antibodies against rubella, herpes virus, toxoplasma, treponema pallidum or cytomegalovirus were not detected. On the basis of these typical findings, the child was diagnosed as having Hypodipsic hypernatremia syndrome. After water metabolism studies were completed, the patient was discharged while receiving oral Desamino-D-Arginine vasopressin (DDAVP) and forced hydration.

## Discussion

The plasma osmolality and its principal determinant, plasma sodium concentration, are normally maintained within a narrow range. This constancy is mostly realized through the coordinated action of osmoreceptors in the hypothalamus which modulate water intake and excretion by regulating thirst and vasopressin secretion [2, 3]. Hypodipsic hypernatremia is a rare disease that associates a lack of thirst and often an abnormal secretion of vasopressin. The disease includes four degrees of severity that are based on the thirst patterns and vasopressin secretion following an infusion of hypersaline solution [3]. Herein, we describe a boy with hypodipsic hypernatremia with cerebral calcifications. The lack of thirst was evident both in basal conditions and in response to osmotic stimuli, irrespective of plasma osmolality. It is a common fact that an increase in plasma osmolality to greater than 300mmol/kg leads to severe thirst; however, this never happened to our patient. Basal vasopressin secretion was evaluated and was inappropriately low for the corresponding level of plasma osmolality (ADH = 1.95 pg/ml) [1]. The underlying disease in this child is uncertain. Developmental delay and intracranial calcifications are consistent with, but not diagnostic of, congenital infections such as cytomegalovirus infection. An extensive investigation of calcium metabolism and an evaluation of somatic features excluded hypoparathyroidism and pseudohypoparathyroidism. In persons having 18 years of age and younger, disordered osmoregulation and chronic hypernatremia have been seen in association with hypothalamic tumors, especially germinoma and gliomas, congenital malformations, histiocytosis and microcephaly [4, 5]. We conclude that, in our patient, sustained or recurrent hypernatremia almost invariably indicates some intrinsic defects in the osmoregulation of thirst and vasopressin. A regimen of forced water intake and receiving DDAVP lead to a satisfactory growth and weight gain.

## Conclusion

Hypodipsic hypernatremia is a rare disorder that is due to a defect in the hypothalamic osmoreceptors for thirst and vasopressin. The disease was apparently due to diffuse brain calcifications and engenders failure to thrive and psychomotor retardation.

## Competing interests

The authors declare no competing interest.
